# From Removal to Selective Control: Perspectives on Predation Management in Spanish Hunting Grounds

**DOI:** 10.3390/ani15192917

**Published:** 2025-10-07

**Authors:** José A. Torres, E. Jorge Tizado, Raquel Castillo-Contreras, Luis F. Villanueva, Carlos Sánchez-García

**Affiliations:** 1 Department of Research, Fundación Artemisan, 13001 Ciudad Real, Spain; jatorres@fundacionartemisan.com (J.A.T.); raquel.castillo@fundacionartemisan.com (R.C.-C.); director@fundacionartemisan.com (L.F.V.); 2 Doctoral Program in Natural Resources and Sustainable Management, University of Cordoba (UCO), 14014 Cordoba, Spain; 3 Department of Biodiversity and Environmental Management, Universidad de León, 24401 Ponferrada, Spain; ej.tizado@unileon.es

**Keywords:** corvids, hunting, management, predation, red fox, restraint traps, wild boar

## Abstract

Historically, predator control has been a frequent practice conducted in the hunting grounds of Spain. After the approval of Law 42/2007 on Natural Heritage and Biodiversity, and the enforcement of international regulations, predator control methods are required to be selective, non-massive, and conducted by trained specialists when traps are used; however, there is a lack of research on the current status of predator management. Data was gathered from 16 regional wildlife departments and from 373 questionnaires from hunting grounds targeting the conservation of small game species. Seven predatory species were included in the regional game species lists, the most frequently controlled being the red fox (*Vulpes vulpes*, 90.4%), wild boar (*Sus scrofa*, 78.3%), and Eurasian magpie (*Pica pica*, 51.5%), with control intensity differing among regions. In total, 87% of the questionnaire respondents declared controlling at least one predator species, while 49.3% employed gamekeepers to perform the control. In all surveyed regions, shooting was authorized, and it was the most frequent control method in hunting grounds for the aforementioned species (ranging from 76 to 100%), while the use of approved restraint methods was allowed in 11 regions but only used in 7 for foxes (8%) and magpies (25%). The control intensity (animals culled/km^2^) for foxes was higher when conducted by full-time keepers, while for magpies and wild boars, it was higher when conducted by hunters. The implementation of habitat management (agricultural and forest measures) that helps to reduce predation was higher in hunting grounds not conducting predator control. Based on our results, we propose a national predation management framework focused on controlling rather than removing predators.

## Simple Summary

Predator control has been historically conducted in the hunting grounds of Spain; after the enforcement of international agreements and regulations, it has been conducted using selective and non-massive methods. In this study, we explored the current predator control regulations in 16 Spanish regions, as well as the management traits at the hunting ground level, using data from 373 questionnaires. Predator control through shooting was legal in all regions, mainly targeting the red fox (*Vulpes vulpes*) and Eurasian magpie (*Pica pica*), and wild boar (*Sus scrofa*) (which was considered a predatory species), while the use of approved restraint traps was allowed in 11 regions (but only used in 7). Predator control was a widespread measure conducted in hunting grounds (87%), and the control intensity was influenced by factors such as who performed the control (hunters or gamekeepers). The current predator management system in Spain is mainly based on predator control, but there is a possibility of shifting towards a “predation management” framework through professionalization and support for habitat management and apex predators.

## 1. Introduction

In countries where small game species are managed for hunting purposes, predator control has historically been a frequent measure conducted among the different game management practices [1]. A significant number of studies have addressed its effectiveness in Europe, North America, and Australasia [2,3]. In Spain, a southwestern European country that holds the stronghold of the red-legged partridge population (*Alectoris rufa*) [4], as well as other small game species such as European rabbit (*Oryctolagus cuniculus*) and Iberian hare (*Lepus granatensis*), research has covered aspects related to the selectivity of methods and their effectiveness when aiming to favor these species of high socio-economic importance [5]. The European rabbit is also a keystone species in the Mediterranean ecosystems of southern Europe [6,7].

Until the 1970s, the majority of predators were perceived as “vermin”; hence, significant efforts were made to eliminate both opportunistic and specialist predators through non-selective and mass methods, through the use of poison and kill-traps that were allowed and even publicly funded [8]. This context started to change with the publication of international and European agreements on nature and wildlife conservation, generally prohibiting massive and non-selective methods (Bern Convention and European Regulation 3254/91). Subsequent agreements on predator control methods were signed afterwards; however, in Spain, the enforcement of these regulations did not start until the 1980s–1990s (Spain joined the EU in 1986). The process was completed in 2007, when the national Law 42/2007 on Natural Heritage and Biodiversity was published, followed by the publication of the predator control guidelines, which had two key points: the use of restraint methods that had surpassed international trapping standards and the training needed to become qualified as a predator control specialist [9]. This training consists of specific courses to ensure that specialists are able to handle and set authorized methods, dispatch target species, and release non-target ones. The candidates must pass a theoretical and practical exam set by the regional administrations.

The most widespread opportunistic predator species in Spain that can be legally controlled are red fox (*Vulpes vulpes*) [10] and Eurasian magpie (*Pica pica*) [11], but during the last decades, there has been a dramatic increase in wild boar (*Sus scrofa*) populations in Spain and Europe [12], which has raised concerns of its impact on many species, especially ground-nesting birds such as red-legged partridge [13] and waterfowl [14]. According to the latest national gamebag survey from the annual review of forest statistics, covering the 2022–2023 hunting season [15], the gamebag figures were 186,283 foxes, 443,714 wild boar, and 275,770 corvids, not distinguishing between corvid species in the regions where several corvids can be removed. None of these predators are considered of conservation concern.

Spain holds a diverse predator community, including apex predators that have been recovering in recent times, such as Spanish imperial eagle (*Aquila adalberti*) [16] and Iberian lynx (*Lynx pardinus*) [17], together with a wide range of raptors, mammals of different size (including canids, felids, mustelids, viverrids, and rodents), reptiles, and even insects [18,19]. In this predator list, stray cats (*Felis catus*) and dogs (*Canis familiaris*) should also be included. Hence, the predator community present in many Spanish hunting grounds may consist of specialist and opportunistic predators, wild anthropophilic species (fox and magpie), and domestic/stray species, together with exotic predators [20,21].

As found by Delibes-Mateos et al. [22] for hunting grounds targeting small game in central Spain, there are economic and social factors driving predator control, as a proportion of managers (mainly from commercial hunting grounds) considered that their economic activity would be difficult to conduct without predator control. In the past, there have been examples of persecution of protected species in hunting grounds after the collapse of the European rabbit populations [23], and the use of non-selective trapping methods led to a poor predator community in some Iberian areas [24,25]. It is true, however, that there has been a change towards tolerance of predators from both hunters and managers, as found for the Iberian lynx, which contributes to the control of mesocarnivores that predate on small game species [26,27].

The majority of the existing research addressing predator control in Spain, conducted before 2007, showed that a high proportion of hunting grounds employed gamekeepers and carried out predator control (mainly targeting red fox and magpie), identifying problems such as the non-selectivity and low efficiency rate of legal methods [28,29], together with the use of illegal methods [22,30,31]. Moreover, the use of poison was still recorded in a non-negligible proportion of hunting grounds in some regions [32], which is perhaps one of the most important wildlife conservation problems in Spain.

However, after almost 20 years since the publication of the aforementioned national law, few studies have evaluated the current predator management traits in the hunting grounds of Spain. It is still necessary to explore questions such as the spatial extent of predation control, its intensity, who performs it (professional gamekeepers or hunters), and what other management measures (e.g., habitat management) may influence predation control. There is evidence that habitat management can help to reduce predation [33,34]; hence, attention should be paid to habitat measures conducted in hunting grounds.

As the management of opportunistic predators is included in the national Game Management Strategy [35] promoted by the Ministry of Agriculture, Fisheries and Food Affairs (MAPA), our findings can be useful for this strategy and for the benefit of the different stakeholders involved. This is particularly relevant in the context of declining small game species alongside the recovery of apex predators. Similar considerations apply to other European countries where predator control is conducted to enhance bird and mammal populations [2,3].

The aims of this research were to (1) review and analyze the current predator control regulations in Spain (including species, periods, and methods used), (2) evaluate the predator management traits in hunting grounds targeting red-legged partridge, and (3) address whether management traits influence control intensity and the implementation of habitat measures.

## 2. Materials and Methods

### 2.1. Study Area and Context

This study was conducted in Spain, where predator control has been conducted historically as part of game management, targeting both big and small game species [8]. In this country, around 43 million hectares (85% of the land) are declared as “hunting ground”, and there are nearly 32,500 hunting grounds, which can be owned by public agencies or by private owners [15], the latter being managed directly by the landowner or by a third party, such as social hunters’ associations. In Spain, it is estimated that there are 577,742 hunters [36].

In Spain, the power on wildlife and game management was delegated from the national government to the regional departments by the end of the 20th century [37]; hence, the legislation about predator conservation and management relies on the regional authorities, which have to comply with international, European, and national regulations [5]. The species, periods, and control methods in each region must be approved by the regional authorities and are published through a yearly hunting regulation (Orden de Vedas).

The majority of predators to be controlled are game species, with the exception of exotic and invasive species. Predator control is conducted either during the regular hunting periods or during specific periods when other species cannot be hunted. In the latter case, a special permit will be required by the regional authority, and predator control will need to be previously included in the hunting management plan (Plan de ordenación cinegético). At the hunting ground level, the decision to conduct predator control is taken by the owner or holder of the hunting rights (Titular del coto), who will submit an application to the regional authority, and the permit may be rejected if conditions are not met. The regional authority will provide quotas depending on predator abundance data and additional factors such as hunting bag records, damage to crops and livestock, and, in some cases, disease transmission risk.

Historically, predator control has been conducted by hunters and gamekeepers and, in some cases, by wildlife wardens. Gamekeepers can be employed full-time or part-time by hunting grounds; the former option is applicable when only certain types of activities are contracted, normally those which hunters may struggle to perform (such as predator control and vigilance). The gamekeepers are trained and must be registered in each region, with some regions distinguishing between different types of gamekeepers according to existing regulations. In all cases, anyone using traps (including hunters) must be certified as a predator control specialist, whereas hunters can control predators through shooting, as long as they hold a gun license, a hunting license, and a permit given by the holder of the hunting rights and the regional administration

### 2.2. Data Collection and Analysis

Data on predator control was gathered from the two key stakeholders: regional governments, who have the power to promote, enforce policies, and provide permissions; and hunting grounds’ managers.

#### 2.2.1. Survey on Predator Control Regulations and Methods Permitted by Regional Authorities

We contacted all the regional game departments to understand the current framework, submitting a questionnaire which had the following questions: (1) Is predator control for game species authorized in your region? (2) Which species of predators can be controlled? (3) Does predator control have specific regulations in your region, including approved restraint methods and certification of predator control specialists? (4) Is predator control authorized in areas with European rabbit overabundance? If not, what kinds of restrictions apply? (5) Which are the authorized control methods? (6) What are the periods in which predator control is authorized? (7) Considering the last 10 years, how many hunting grounds have been authorized on a yearly basis to conduct predator control? (8) How many people were certified as predator control specialists in the last 10 years? The last question is for those regions in which specialists are certified.

The information gathered from the questionnaire was complemented by phone interviews to gain additional data and solve further queries.

#### 2.2.2. Questionnaire on Predation Control Conducted in Hunting Grounds

We aimed to gather data on the game management conducted in hunting grounds dedicated to the conservation and hunting of the red-legged partridge (hereafter referred to simply as partridge), one of the most important small game species in Spain [38]. Previous research confirmed that the management of this species includes predator control at different levels, depending on the economic interest of the grounds [19,22].

The questionnaire used in this study was based on a previous project evaluating the habitat management targeting the European turtle dove (*Streptopelia turtur*) and other small game species in Spain and Europe [39]. The questionnaire was developed in close collaboration with hunters, game managers, hunters’ federations, and landowners, who belonged to an existing network of hunters taking part in a volunteer game and wildlife monitoring project (www.observatoriocinegetico.org, accessed on 5 May 2025). Before submitting the questionnaire to the contacts, it was piloted with 13 members of the monitoring project to ensure correct wording, taking on average 12 min to complete the questionnaire when the information was ready to be entered.

The questionnaire was implemented in Google Forms © and delivered directly to the contacts. At the beginning of the questionnaire, there was a summary to explain the aims of the study, including information about the privacy settings and rights of the participants. In compliance with the Spanish data protection law, all participants had to indicate explicitly that they agreed to participate in the questionnaire. The questionnaire had six sections: (1) basic data of the hunting ground (which was the unit of analysis), (2) habitat management measures, (3) predator control, (4) partridge hunting, and (5) partridge releasing (Appendix A).

The survey was conducted between 2022 and 2024, and the participants were asked to provide data for two consecutive hunting seasons: 2022–2023 and 2023–2024. 

#### 2.2.3. Statistical Analyses

We calculated mean regional control intensity values (animals culled/km^2^) for the red fox and wild boar using the latest data available from the Spanish annual review of forest statistics (covering the 2022–2023 season) [15]. It was not possible to calculate the values for magpies from the annual review because in some regions the magpie gamebag was merged with other corvids. 

As the study used data from two consecutive seasons, in those hunting grounds that submitted the questionnaire for two seasons, the information was merged into the second season to avoid pseudoreplication. The implementation of predator control, habitat measures, and partridge releasing was categorized as “conducted” when they were implemented at least during one season, and for each predator species, the median control intensity in each hunting ground was calculated across the two seasons. All predator control methods conducted during the two seasons were considered. 

Chi-squared and Fisher’s exact tests were performed to assess whether the implementation of habitat measures that could reduce predation in hunting grounds (agricultural and forest measures) differed significantly between those conducting predator control and those not. A test was conducted considering the responsibility of gamekeeping (conducted by hunters, part-time, or full-time gamekeepers) and partridge releasing (distinguishing between those releasing and those not).

A Kruskal–Wallis test was applied to address whether the control intensity of red fox, magpie, and wild boar (animals culled/km^2^) conducted in the hunting grounds was affected by gamekeeping responsibility, followed by a post hoc Dunn’s test. All analyses were conducted in R version 4.4.1 [40]. 

Density maps of control intensity per region were created using Jenks Natural Breaks [41], using data from the annual review and questionnaires from the hunting grounds. This method allows the identification of natural groupings in the data to minimize the variance within each class and maximize the variance between classes [42]. 

## 3. Results

### 3.1. Survey on Predator Control Regulations and Methods Permitted by Regional Authorities

Predator control was allowed in all 16 contacted regions, and seven different species were included in the game species lists, the most frequent being red fox, magpie, and wild boar (allowed in all peninsular regions), but also other corvids and two gull species in northern Spain (Appendix B). Several regions pointed out that the control of stray cats and dogs had become illegal in the last decade, owing to regional and national animal welfare regulations (such as the national Law 7/2023). Out of 16 regions contacted, 11 followed national regulations and 5 had specific regional regulations.

Predator control through shooting was authorized in all regions, while the use of restraint traps was not allowed in the Atlantic regions (with the exception of Navarra), and in Islas Baleares, only a selective cage trap for stray cats was authorized. In the peninsular regions where restraint traps were authorized (*n* = 10), three types of snares for the red fox were allowed, including non-locking Spanish snare, Wisconsin and Collarum^®^ (Wildlife Control Supplies EU, Tarragona, Spain), and a cage trap for corvids (Jauteco, Castellón, Spain, see descriptions in [29,43,44]), with additional traps in some regions for fox (Belisle Selective^®^, Wildlife Control Supplies EU, Tarragona, Spain) and corvids (Larsen and Ladder traps) (Appendix C).

**Figure 1:**
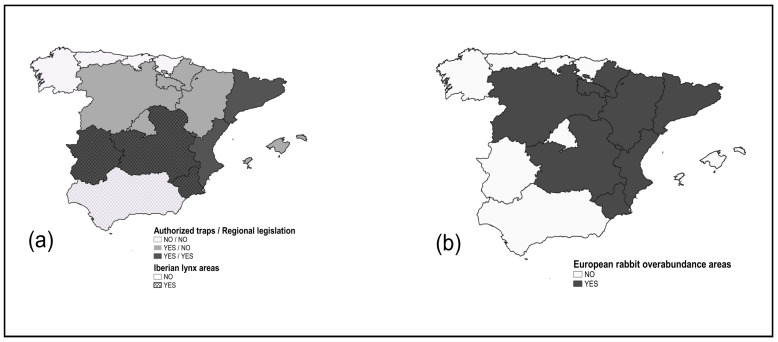
Map of Spain: (**a**) the authorization of restraint traps for predator control, existence of regional predator control regulations, and restrictions within the Iberian lynx range; (**b**) red fox control restrictions in hunting grounds within emergency game areas.

In those regions where breeding populations of Iberian lynx occurred (Castilla-La Mancha, Extremadura, and Murcia), specific restrictions applied during the post-breeding periods of the species in expansion areas (Figure 1a). For example, in Extremadura, the non-locking Spanish snare and Wisconsin snare were not allowed, and the magpie cage traps had to be positioned > 1 m above ground to enhance selectivity and prevent accidental lynx cub captures. In Castilla-La Mancha, the minimum loop size of Wisconsin snares had to be increased from 6.5 cm to 9 cm.

In eight regions, red fox control was restricted in hunting grounds within emergency game areas affected by European rabbit overabundance, as fox predation may help to reduce rabbit overabundance; other rabbit hunting methods were allowed (Figure 1b).

Predator control through shooting was authorized in all regions during the regular hunting season (in the majority, spanning from October to February), and also during August and September for red fox and magpie. In the majority of regions, restraint traps were authorized in spring and early summer (coinciding with the small game species breeding periods), but some regions allowed their use in winter and autumn periods (Appendix D).

Nine regions provided data on the number of control permits approved per year, with Castilla-La Mancha standing out, with a yearly mean of around 750 (including shooting, traps, and both), whereas in the other regions, the number of permits was much lower or nonexistent. Although restraint traps were allowed in Aragón and Castilla y León, no authorizations were given during the study period. The mean number of people who were certified as predator control specialists per year and region ranged from 5 to 368, with the highest values recorded in regions where no control permits were approved in recent years (Comunidad Valenciana and Cataluña) (Table 1).

### 3.2. Questionnaire on Predation Control Conducted in Hunting Grounds

**Figure 2:**
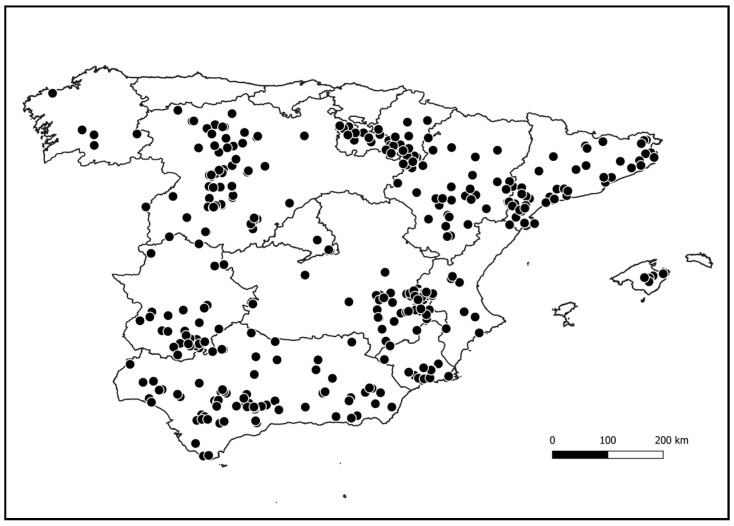
Locations of the 373 hunting grounds that submitted the questionnaires.

A total of 373 valid questionnaires were received (Figure 2), and 86.8% of respondents reported conducting predator control (*n* = 324). The majority of the grounds targeted the management and conservation of small game species (55.9%), in combination with big game in 38.4% of the grounds, and the remaining 5.7% targeted only big game.

**Figure 3:**
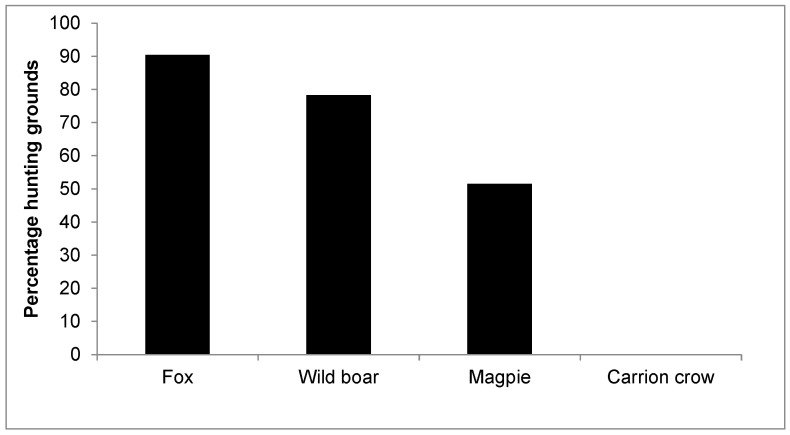
Percentage of hunting grounds (*n* = 324) controlling each predator species.

The fox was controlled in 90.4% of the grounds, followed by the wild boar (78.3%) and the magpie (51.5%), with only one hunting ground declaring the control of carrion crows (*Corvus corone*) (Figure 3). The most frequent pattern was to control fox, wild boar, and magpie (40.4% of grounds), or fox and wild boar (31.17%), while other combinations were less frequent. Twenty-one hunting grounds (6.5%) declared that stray cats were removed when it was allowed.

**Figure 4:**
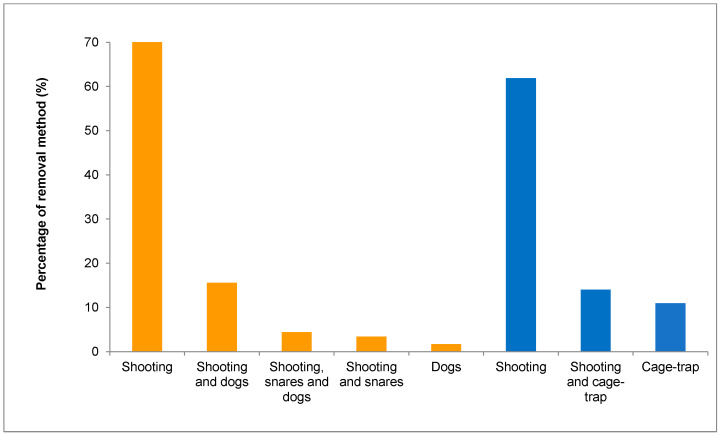
Percentage of hunting grounds using the different control methods allowed for red fox (in orange, *n* = 293) and magpie (in blue, *n* = 190).

For fox, the most common control methods were shooting (71%) and shooting with terrier dogs (15.6%), while for magpie, shooting accounted for 61.9% of the grounds, followed by shooting and cage-trapping (14%) (Figure 4). For wild boar, the only control method declared was shooting.

The control intensity (number of animals culled/km^2^) per hunting ground for red fox (*n* = 274) ranged from 0.01 to 16.13 (median = 0.56, lower quartile = 0.31, upper quartile = 1.24), and for magpie (*n* = 142), it ranged from 0.01 to 19.90 (median = 0.68, lower quartile = 0.19, upper quartile = 1.73). For wild boar (*n* = 229), the control intensity ranged from 0.03 to 18.34 (median = 0.74, lower quartile = 0.28, upper quartile = 1.70).

**Figure 5:**
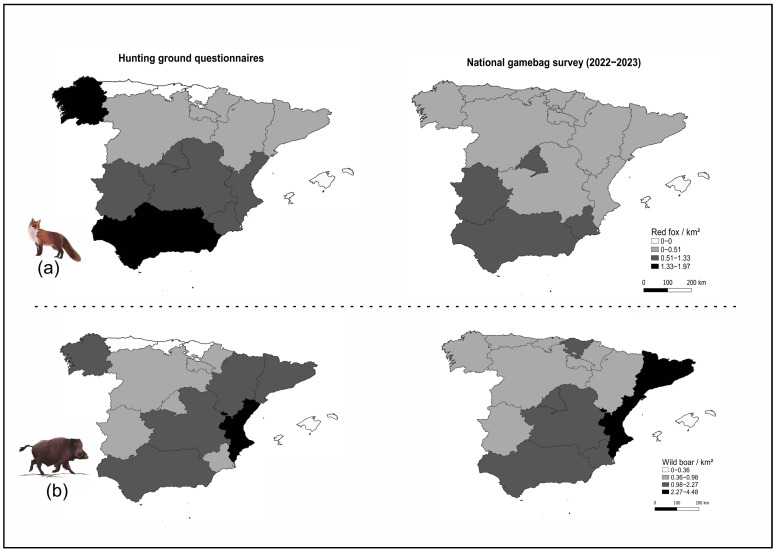
(**a**) Mean regional values of control intensity (number of animals culled/km^2^) for red fox using the questionnaires (left) and data from the national gamebag survey (Anuario de Estadística Forestal) for the 2022—2023 hunting season, and (**b**) the same information for wild boar.

At the regional level, the mean fox control intensity calculated from the questionnaires was higher than the mean values from the national gamebag survey, with higher intensity in grounds from southern Spain (notably Andalucía and Galicia) and other regions in central and eastern Spain (Castilla-La Mancha and Comunidad Valenciana). For wild boar, a higher intensity was recorded when the values were calculated using the national gamebag survey in Cataluña and Murcia, with higher values also recorded in the Atlantic regions (with the exception of Galicia), for which no questionnaires were available (Figure 5). 

When considering the habitat management measures that could reduce predation in the hunting grounds, 51% of respondents declared to conduct agricultural or forest measures; in those grounds that did not control predators (*n* = 49), the percentage was significantly higher (61.01%) compared to those that controlled predators (49.58%) (χ^2^ = 13.21, *p* = 0.001). In the former, forest management was the most frequent measure (42.37%), while in the latter, it was agricultural measures (28.08%). 

Out of 373 grounds, 49.3% declared to employ gamekeepers, either full-time (15.3%) or part-time (34%), while the remaining 50% of gamekeeping (including tasks related to predator control) was conducted by hunters. The hunting grounds conducting control employed full-time gamekeepers in a higher proportion compared to grounds not conducting control, in which part-time keepers were employed at a higher rate (*p* = 0.025).

The Kruskal–Wallis test showed significant differences in control intensity (animals killed/km^2^) among the three types of gamekeeping for red fox (*p* < 0.001), magpie (*p* < 0.001), and wild boar (*p* = 0.008). For red fox, the post hoc Dunn’s test showed that a higher control intensity was recorded in the hunting grounds employing full-time gamekeepers compared to part-time gamekeepers and hunters (*p* < 0.001), while for magpie, a higher control intensity was achieved by hunters compared to full-time (*p* = 0.005) and part-time gamekeepers (*p* < 0.001). For wild boar, the control intensity was marginally higher when conducted by hunters compared to full-time gamekeepers (*p* = 0.049) (Table 2). The proportion of respondents declaring releasing farm-reared partridges was 27%, and the implementation of predator control was not affected by the release of partridges (χ^2^ = 0.37, *p* = 0.541)

## 4. Discussion

Our results confirm that the control of predators was allowed in all the surveyed Spanish regions, with differences in the species authorized to be controlled, methods, and periods. When considering hunting grounds targeting the management of partridges, predator control remained a frequent measure performed by hunters and gamekeepers. The information gathered shows that the current scenario is mainly based on the selective control of a reduced list of opportunistic predators, but with the possibility of moving forward into a predation management framework incorporating new elements.

Although predator control was allowed in all regions, not all of them had specific regulations. Moreover, approved restraint traps were authorized in 11 regions (including a cage trap for stray cats in Islas Baleares, Murcia, and Extremadura), but were only used in 7, while in 5 regions they were banned. The lack of regional regulations did not prevent predator control through shooting or trapping, as national regulations and guidelines apply in all regions, but we understand that the possibility of using restraint traps is an opportunity for trained professionals to become predator control specialists, which is one of the aims of the current predator control framework [9]. In this way, every year, a significant number of people (i.e., gamekeepers, wildlife wardens, and hunters) become qualified in some regions; hence, there is an interest in using restraint traps.

In Extremadura and Castilla-La Mancha, where the Iberian lynx has experienced a sharp population increase in the last 20 years [17], the majority of restraint methods were allowed, with some restrictions applied to avoid accidental lynx cub captures. This confirms that it is possible to conduct selective predator control in areas holding apex predators; in this case, it is a species that helps to increase the abundance of small game through the suppression of mesocarnivores [26,27].

Seven different predator species were included in the regional game lists, the most frequent being red fox, magpie, and wild boar. Opportunistic predators are widely distributed across the Iberian Peninsula, which may include in their diet small game but also other species [10,11,45,46]. The case of the wild boar is particular, because in the regulations it is considered a big game species and not a predator in the strict sense, but in all the regions, there were several hunting types allowed to control its populations, such as driven-hunting, sit-and-wait, and even trapping when hunting is not effective or difficult to implement [47,48]. Thus, the regulations have adapted to a new scenario in which wild boar may act as a game species of high socioeconomic importance in some areas, but also as an opportunistic predator to be controlled, including in contexts of crop damage [49].

We understand that the inclusion of carrion crows and jackdaws (*Coloeus monedula*) in the game list of nine and three regions, respectively, can be explained not only by control purposes in hunting grounds, but also by the need to remove animals in contexts of crop and livestock damage, though in Spain there is a lack of research on this topic [50,51]. The same applies to the two gull species, which can be controlled in six regions (including five coastal ones).

Stray cats and dogs were not included in the predators’ list; hence, it is not possible to remove these species from natural areas (with few exceptions when endangered species are at risk), despite free-roaming cats being a proven threat for wildlife worldwide [52,53]. This is a result of the publication of regional and national laws dealing with animal welfare, in which these species are protected, though the national Law 7/2023 allows the control of stray cats from natural areas (called “community cats”) through selective and non-lethal methods. There is already a selective restraint trap for cats that has surpassed the trapping standards [54] and has been used successfully in research projects conducted in Mediterranean habitats with a diverse mesocarnivore community [55].

The information gathered from the questionnaires on predator control in hunting grounds showed that the majority of participants (87%) controlled predators, with red fox being the most controlled, followed by wild boar and magpie. This is partially in agreement with the review of Díaz-Ruiz [30] and other studies conducted in Spain more than 10 years ago, in which the majority of control efforts in small game hunting grounds targeted red fox and magpie [56,57]. As the wild boar may have detrimental effects on partridges and wild rabbits [58], and their populations are expanding, it is not surprising that hunters and gamekeepers managing small game species would now consider the wild boar as a predator and act accordingly.

Studies conducted in the UK have shown that lethal fox control remains a frequent tool conducted in hunting grounds targeting gamebirds [59], and the same applies for corvids [60]. In other European countries, it is difficult to estimate the proportion of grounds controlling predators, though the gamebag data suggests that a high proportion may conduct fox and wild boar control (using lethal and non-lethal methods) to prevent and reduce damage to other animals, crops, and disease transmission [12,61]. In this way, controlling these species is one of the tools used when aiming to eradicate rabies and African swine fever, though they should be combined with others, including non-lethal methods and carrion management [62,63,64]. Spain is currently free from these diseases, but if fox and wild boar were to be controlled for disease reasons, our findings could help policy makers and practitioners develop prevention and eradication strategies.

When we compared the control intensity values at the regional scale from hunting grounds to the ones from the national gamebag survey (Figure 5), the values were higher for red fox in the hunting grounds and partially similar for the wild boar. In the case of the fox, this could be attributed to the fact that in the hunting grounds participating in this research, there was a higher interest in favoring small game (94% of the grounds targeted these species) than in the average Spanish hunting ground, and consequently, efforts would be implemented to increase the control intensity.

As expected, the most widespread method to control red fox and magpie was shooting (in the case of fox, often conducted simultaneously with terrier dogs), though the use of restraint traps was not negligible. This is not surprising, because shooting can be conducted without the need to be qualified as a predator control specialist, and not all regions allowed traps. In relation to this, the control intensity varied significantly among hunting grounds, likely explained by the different management traits, as reported by Delibes-Mateos et al. [22]. The higher red fox control intensity in hunting grounds employing full-time gamekeepers could be explained by the higher effort invested by the gamekeepers or the higher efficiency of the methods. In contrast, the magpie and wild boar control intensity was higher when conducted by hunters, which can be attributed to the higher intensity achieved in hunting for both species (in wild boar through driven-hunting and sit-and-wait), where the involvement of gamekeepers is not mandatory [64]. Further research is needed on this topic.

The habitat management data gathered in the questionnaire showed a similar rate of implementation compared to other studies conducted in Iberia [39]; 51% of the grounds employed resources in habitat measures that may reduce predation. The higher rate of implementation in the grounds where no control was conducted points towards the idea that, in some cases, habitat management would be the priority rather than predator control, though we do not know the reasons behind this decision. In Spain, some authors argue that habitat management is the key tool when aiming to recover red-legged partridge and wild rabbit [33,65].

In perspective, the control of predators has changed dramatically in Spain in recent decades as a consequence of the enforcement of international agreements for control methods [5], resulting in a significant number of people becoming qualified as predator control specialists and, at the same time, a declining use of illegal methods in hunting grounds [66]. As habitat management and the increased occurrence of apex predators in hunting grounds (often persecuted in the past) reduce predation, the challenge now is how to incorporate and promote these two key elements in the current scenario.

## 5. Conclusions and Management Recommendations

The current predator management in Spain is mainly based on the selective control of opportunistic predators, but there is a possibility to move towards a predation management framework which could be incorporated within the current national Game Management Strategy.

Specific regulations should be approved in all regions as a way to promote the professionalization of predator control and the use of restraint traps not only in hunting grounds, but also in areas where protected species are negatively affected by generalist predators.

Together with predators currently included in the game lists, there is an urgent need to facilitate the control of stray cats through selective and non-killing methods, as they cannot be considered a protected species in hunting grounds or protected areas (in fact, the Law 7/2023 allows their control). If scientific evidence supports the need to control a given predator species, policy makers should be ready to make decisions accordingly.

Although current regulations have shifted towards the facilitation of control actions through shooting for certain species (such as the wild boar), and new technologies have been incorporated to improve practical aspects, predator control actions could benefit from the use of camera traps, automated sensor traps, and mobile phone applications for checking daily traps. In this way, regular training for specialists should be offered with regular update courses.

Habitat management interventions (i.e., agricultural and forest measures) should be supported by regional administrations to increase the proportion of grounds and surface area dedicated to these measures. To consolidate the recovery of apex predators into new areas, the conservation of these species should involve hunters and game managers from early stages, as has already been conducted for the Iberian lynx.

Predator control already requires the monitoring of the actions conducted and animals bagged, but it should also incorporate the monitoring of predators (both controlled and not), and their potential prey. Current digital tools used by hunters in voluntary monitoring schemes could help to gather data for subsequent analyses (www.observatoriocinegetico.org, accessed on 5 May 2025), and in the coming years, it is expected that gamebag recording will be conducted using mobile phone applications. 

## Figures and Tables

**Table 1:** Mean number of control permits and certified predator control specialists per year and region, showing in brackets the period for which data was available.

Region	Control Permissions (Period)	Certified Predator Control Specialists
Castilla y León	843 (2014–2024) *	–
Castilla-La Mancha	745.8 (2020–2023) **	34.8 (2020–2023)
Navarra	76 (2014–2023) *	5 (2014–2023)
Madrid	50 (2014–2023) **	60 (2014–2023)
Extremadura	45 (2021–2023) **	55.3 (2014–2023)
Aragón	43.4 (2014–2020) ***	–
La Rioja	22.3 (2014–2023) **	–
Murcia	2 (2022–2023) **	16.3 (2021–2023)
Com. Valenciana	–	368.2 (2015–2023)
Cataluña	–	98 (2014–2023)

* Only considering shooting; ** both shooting and restraint traps; *** restraint traps were allowed, but no permits were given.

**Table 2:** (a) Percentage of respondents conducting agricultural and forest measures considering the control of predators; (b) percentage of respondents considering the gamekeeping responsibility in grounds conducting control (*n* = 324); and (c) control intensity (animals killed/km^2^) for red fox, magpie, and wild boar (median), depending on who holds the gamekeeping responsibility.

(a) Habitat Management (%)	No Measures	Agricultural	Forest		
All grounds	48.80	27.38	23.80		
No control	38.98	18.64	42.37		
Control	50.41	28.80	20.77		
(b) Type of Gamekeep. (%)	Hunters	Part-time gamekeep.	Full-time gamekeep.		
No control	51.02	44.89	4.08		
Control	50.61	32.4	16.97		
(c) Control intensity (*n* culled/km^2^)	Hunters	Part-time	Full-time	H	*p* value
Red fox	0.42	0.51	0.74	47.6	<0.001
Magpie	0.00	0.23	0.00	14.5	<0.001
Wild boar	0.65	0.41	0.09	7.1	0.008

## Data Availability

The data presented in this study are available upon request from the corresponding author.

## Appendix A

List of questions submitted to the hunting grounds.

(1) Basic data of the hunting ground (unit of analysis) included surface area, responsibility of the respondent concerning game management, agricultural and forest management, presence of fences, and type of gamekeeping. The latter questions allowed us to address whether hunters or gamekeepers (employed part-time or full-time) conducted predator control.
(2) Habitat management measures: food and water provision (water troughs and ponds), agricultural management for game and wildlife (including game crops, management and conservation of stubbles and fallows), agri-environmental measures funded by public schemes (measures similar to the agricultural ones), and forest/shrub management (including forest and shrub clearing). Respondents were asked whether the management was implemented (or not), the number of measures or the total surface involved, the sources of funding for the management, and practical questions (such as the types of seeds in the agricultural measures).
(3) Predator control: species, methods (hunting and traps), and number of animals killed.
(4) Partridge hunting: types of hunting, harvesting levels, and restraint measures when implemented.
(5) Partridge releasing: number of partridges released.

## Appendix B

**Table A1:** List of predator species included in the regional quarry list.

Region	Red Fox	Wild Boar	Magpie	Carrion Crow	Jackdaw	Others
	*Vulpes vulpes*	*Sus scrofa*	*Pica pica*	*Corvus corone*	*Corvus monedula*	
Andalucía	Yes	Yes	Yes	No	Yes	
Aragón	Yes	Yes	Yes	Yes	No	
Asturias	Yes	Yes	Yes	Yes	No	Yellow-legged gull *
Cantabria	Yes	Yes	Yes	Yes	No	Yellow-legged gull *
Castilla La Mancha	Yes	Yes	Yes	Yes	No	
Castilla y León	Yes	Yes	Yes	Yes	No	
Cataluña	Yes	Yes	Yes	No	No	
Comunidad Valenciana	Yes	Yes	Yes	No	No	
Comunidad de Madrid	Yes	Yes	Yes	Yes	No	
Extremadura	Yes	Yes	Yes	No	Yes	
Galicia	Yes	Yes	Yes	No	Yes	Yellow-legged gull *
Islas Baleares	No	No	No	No	No	
La Rioja	Yes	Yes	Yes	Yes	No	Black-headed gull **
Navarra	Yes	Yes	Yes	Yes	No	
País Vasco ***	Yes	Yes	Yes	Yes	No	Yellow-legged gull *
Región de Murcia	Yes	Yes	Yes	No	No	Yellow-legged gull *

* *Larus michahellis*, ** *Chroicocephalus ridibundus*, *** Only includes the province of Guipúzcoa.

## Appendix C

**Table A2:** List of authorized restraint traps for red fox and corvids per region.

	Red Fox				Corvids		
Region	Non-Locking Spanish Snare	Wisconsin Snare	Collarum	Belisle Selective	Cage-Trap	Larsen Trap	Ladder Trap
Aragón	Yes	Yes	Yes	No	Yes	No	No
Castilla La Mancha	Yes	Yes	Yes	Yes	Yes	Yes	Yes
Castilla y León	Yes	Yes	Yes	No	Yes	No	No
Cataluña	Yes	Yes	Yes	Yes	Yes	No	No
Comunidad Valenciana	Yes	Yes	Yes	No	Yes	No	No
Madrid	Yes	Yes	Yes	Yes	Yes	Yes	Yes
Murcia	Yes	Yes	Yes	Yes	Yes	Yes	Yes
Extremadura	Yes	Yes	Yes	Yes	Yes	No	No
La Rioja	Yes	Yes	Yes	Yes	Yes	No	No

## Appendix D

**Table A3:** Months when the use of restraint traps for red fox and magpie is authorized per region.

**Red fox**	**Jan**	**Feb**	**Mar**	**Apr**	**May**	**Jun**	**Jul**	**Aug**	**Sep**	**Oct**	**Nov**	**Dec**
Castilla La Mancha												
Comunidad de Madrid												
Región de Murcia												
Cataluña												
Extremadura												
Comunidad Valenciana												
La Rioja												
**Magpie**	**Jan**	**Feb**	**Mar**	**Apr**	**May**	**Jun**	**Jul**	**Aug**	**Sep**	**Oct**	**Nov**	**Dec**
Castilla La Mancha												
Comunidad de Madrid												
Región de Murcia												
Cataluña												
Extremadura		*	*									
Comunidad Valenciana												
Comunidad Foral de Navarra												
La Rioja												

* Cage traps targeting magpies are allowed to be used, with restrictions in areas where the Iberian lynx is expanding.
